# A Comparative Analysis of Low-Density Lipoprotein Cholesterol (LDL-C)-Lowering Activities of Bempedoic Acid, Inclisiran, and PCSK9 Inhibitors: A Systematic Review

**DOI:** 10.7759/cureus.69900

**Published:** 2024-09-22

**Authors:** Yazhini Rajendran, Madhumita Nandhakumar, Madhavi Eerike, Nikhila Kondampati, Kalpana Mali, Leo F Chalissery, Venu Gopala R Konda, Uma Maheswari Nagireddy

**Affiliations:** 1 Pharmacology, Postgraduate Institute of Medical Education and Research, Chandigarh, IND; 2 Pharmacology, All India Institute of Medical Sciences, Raipur, Raipur, IND; 3 Pharmacology, All India Institute of Medical Sciences, Bibinagar, Bibinagar, IND; 4 General Medicine, GEM Hospital, Chennai, Chennai, IND; 5 Pharmacology, TRR Institute of Medical Sciences, Hyderabad, IND; 6 Pharmacology, Government Medical College, Rajamahendravaram, Rajamahendravaram, IND

**Keywords:** apolipoprotein-b, bempedoic acid, dyslipidemia, inclisiran, ldl-c, pcsk9 inhibitors, serious adverse events (saes)

## Abstract

Newer drugs, such as bempedoic acid, inclisiran, alirocumab, and evolocumab have recently been introduced for dyslipidemia. This systematic review aims to perform a comparative analysis of these drugs' low-density lipoprotein cholesterol (LDL-C)-lowering activities. The PubMed database was utilized to search for randomized controlled trials. Articles were screened and selected based on specific inclusion and exclusion criteria. The primary outcome of this review is to compare the percentage reduction of LDL-C and apolipoprotein-B, along with the number of reported serious adverse events (SAEs) in trials specific to each drug. A total of 14 studies were included, four for bempedoic acid and alirocumab and three for evolocumab and inclisiran. The maximum percentage reduction in LDL-C and apolipoprotein-B from baseline to 12 weeks was observed with alirocumab, administered at 150 mg subcutaneously twice weekly for 12 weeks, achieving reductions of 72.4% and 57.9%, respectively. Lesser reductions were observed with bempedoic acid, administered at 180 mg once daily orally for 12 weeks. The highest number of SAEs were reported with bempedoic acid (216, 10%) and inclisiran (181, 11%; 175, 11%). This systematic review showed that alirocumab achieved the greatest reductions in LDL-C and apolipoprotein-B and a better safety profile. Newer LDL-C-lowering drugs show promise in improving lipid profiles, patient compliance, and safety. However, these findings are not conclusive, as other factors also influence treatment choice.

## Introduction and background

Dyslipidemia is a key risk factor for atherosclerotic cardiovascular diseases (ASCVDs), including coronary heart disease, ischemic cerebrovascular disease, and peripheral vascular disease, and can markedly worsen the prognosis if not properly managed. It is characterized by elevated levels of serum total cholesterol, low-density lipoprotein cholesterol (LDL-C), and triglycerides (TG), along with decreased levels of serum high-density lipoprotein cholesterol (HDL-C) [1,2]. Apolipoprotein-B serves as a component of all atherogenic particles, including very high-density lipoprotein (VLDL), intermediate-density lipoprotein, LDL, and lipoprotein(a). Higher apolipoprotein-B levels correlate with greater amounts of these atherogenic particles, thereby increasing the risk of plaque formation and cardiovascular events such as heart attacks and strokes [3].

LDL-C is the primary cause of most cardiovascular disease (CVD) events [2]. According to the World Health Organization, approximately 39% of the global adult population has elevated blood cholesterol levels, with a slightly higher prevalence observed in women (40%) compared to men (37%) [4].

Evidence from multiple prospective, randomized studies has demonstrated that patients achieving the lowest LDL-C levels have the lowest risk of future major adverse cardiovascular events (MACEs), without significant safety concerns or adverse events, even at very low LDL-C levels [5].

The primary drugs used to treat dyslipidemia are statins, fibrates, bile acid sequestrants, niacin, etc. The following are the recently approved drugs to treat dyslipidemia. Proprotein convertase subtilisin/kexin type 9 (PCSK9) inhibitors are monoclonal antibodies that enhance LDL receptor availability on hepatocytes, reducing LDL-C levels. These drugs, such as alirocumab and evolocumab, are endorsed for statin intolerance or atherosclerotic CVD and are administered every two to four weeks, showing a 27% reduction in heart attack risk [6].

Inclisiran, a novel small interfering RNA (siRNA), inhibits PCSK9 protein translation in hepatocytes, promoting LDL receptor presence on cell surfaces and subsequent LDL-C uptake, lowering serum LDL-C levels. Administered subcutaneously, it shows sustained near 50% LDL-C reduction with two doses (day 1, day 90), followed by injections every six months, offering a promising therapeutic option for dyslipidemia with less frequent dosing [7].

Bempedoic acid has been recently approved for lipid management in patients with ASCVDs or heterozygous familial hypercholesterolemia (HeFH). Its activity is limited to the liver; hence, the risk of myopathy is lower with bempedoic acid compared to statins. Bempedoic acid’s unique mechanism complements other lipid-lowering agents, making it suitable for combination therapy [8].

Comparative efficacy analyses of non-statin lipid-lowering therapies in patients with hypercholesterolemia at increased cardiovascular risk have demonstrated that inclisiran, evolocumab, and alirocumab provide superior LDL-C reduction compared to placebo, bempedoic acid, and ezetimibe. Inclisiran's efficacy is comparable to that of alirocumab [9].

A network meta-analysis assessed the non-statin lipid-lowering therapies, along with maximally tolerated statins, focusing on LDL-C reduction at weeks 12 and 24, and changes in non-HDL cholesterol and apolipoprotein-B at week 12, in which evolocumab (140 mg biweekly/420 mg monthly) and alirocumab (150 mg biweekly) were consistently the most effective in achieving guideline-recommended lipid targets [10]. Recently, various systematic reviews and meta-analyses were conducted with the newer hypolipidemic drugs, which focused mainly on MACE and all the lipid profile parameters [11,12].

This systematic review was conducted to examine the percentage reduction in LDL-C and apolipoprotein-B and safety outcomes with bempedoic acid, evolocumab, alirocumab, and inclisiran at 12 weeks, as these are the specific LDL-C-lowering agents.

## Review

Materials and methods

Eligibility Criteria

This systematic review included randomized controlled trials involving patients of all age groups diagnosed with hypercholesterolemia, regardless of the comorbidities and underlying etiology, who are on stable statin therapy or maximally tolerated statin therapy and should have received bempedoic acid, inclisiran, or PCSK9 inhibitors as intervention. The included studies should have a comparator of standard treatment or placebo. We excluded observational studies, editorial letters, review articles, conference abstracts, quasi-experimental studies, case reports, case series, and animal studies.

Information Sources

The PubMed, PubMed Central, and MEDLINE databases were utilized to search for relevant studies. The search terms used in this study are attached in the supplementary appendices.

Selection Process

Using the search strategy, the titles and abstracts of relevant studies without any language restriction were screened by two independent authors based on the inclusion and exclusion criteria. Additionally, studies with inadequate data or inaccessible full-text articles, follow-up studies, conference proceedings, review articles, non-human studies, duplicate publications, or multiple reports of the same study were excluded from our review. To determine the eligibility, the full text of the articles was finally screened by the two independent authors. Since it is a qualitative analysis, the protocol of this systematic review was not registered with the PROSPERO network.

Data Extraction Process

Two authors independently extracted data using a standardized data extraction spreadsheet. The data extracted includes the general characteristics of the articles, population, intervention, comparison group, and outcome of interest (according to the study objectives). During data extraction, no simplifications or assumptions were made. The risk of bias assessment was conducted using the Cochrane Risk of Bias (ROB) tool (The Cochrane Collaboration, London, England, UK) for this study.

The efficacy parameters evaluated in the study were percentage reductions in LDL-C and apolipoprotein-B from baseline to 12 weeks. The safety parameters measured were serious adverse events (SAEs) reported.

Results

**Figure 1:**
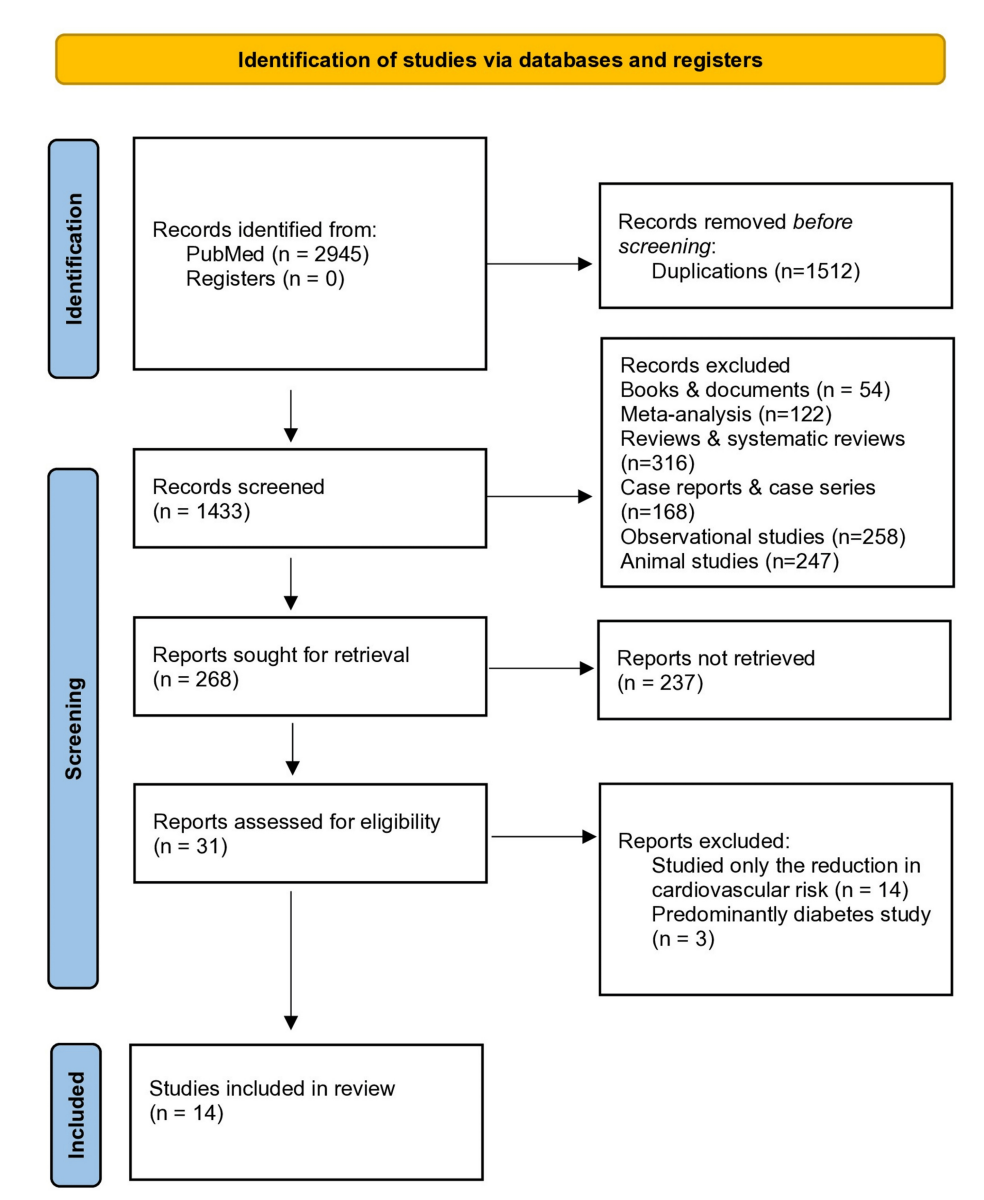
PRISMA flow diagram PRISMA: Preferred Reporting Items for Systematic Reviews and Meta-Analyses

Figure 1 depicts the data extraction details with the help of the Preferred Reporting Items for Systematic Reviews and Meta-Analyses (PRISMA) flowchart. The total number of articles retrieved from PubMed using the search terms as stated in the methodology was 2945 as of April 30, 2024, comprising 439 articles on bempedoic acid, 1126 articles on evolocumab, 98 articles on alirocumab, and 393 articles on inclisiran. Upon screening, a total of 122 meta-analysis reports, 316 reviews and systematic reviews, 54 books and documents, 168 case reports and case series, 258 observational studies, and 247 animal studies were excluded. A total of 268 articles were sought for retrieval, out of which 31 were assessed for eligibility, and a total of 14 articles were included in this systematic review.

Table 1 provides the baseline characteristics of the studies, such as study duration, study design, sample size, significant inclusion and exclusion criteria, primary and secondary efficacy endpoints, and conclusion of the included studies.

**Table 1:** Baseline characteristics of the studies included CLEAR: cholesterol-lowering via bempedoic acid, LDL-C: low-density lipoprotein cholesterol, PCSK9: proprotein convertase subtilisin kexin type 9, P/O: orally, Q2W: every two weeks, QM: every month, Q4W: every four weeks, ASCVD: atherosclerotic cardiovascular disease, eGFR: estimated glomerular filtration rate, BMI: body mass index, CVD: cardiovascular disease, CV: cardiovascular, LMT: lipid-modifying therapy, HbA1C: hemoglobin A1C, MI: myocardial infarction, T2DM: type 2 diabetes mellitus, NYHA: New York Heart Association, HeFH: heterozygous familial hypercholesterolemia, LLT: lipid-lowering therapy, LDL: low-density lipoprotein, HDL-C: high-density lipoprotein cholesterol, HDL: high-density lipoprotein, TG: triglycerides, VLDL: very high-density lipoprotein, CRP: C-reactive protein, apoB: apolipoprotein-B, hsCRP: high-sensitivity C-reactive protein, USA: United States of America, UK: United Kingdom

Study no.	Author, study duration, study year, country	Study design/trial name	Sample size	Intervention details	Inclusion and exclusion criteria	Primary and secondary outcomes	Conclusion of the study
1	Ballantyne et al. [13], 12 weeks, 2016, USA	Phase 2b, multicentric, double-blind, randomized controlled trial	134, test: 89, placebo: 45	Bempedoic acid 120 mg, 180 mg P/O, or placebo once daily in addition to ongoing statin therapy for 12 weeks	Inclusion: hypercholesterolemic patients of both sexes aged 18-80 years and on stable statin therapy after washout of lipid-regulating agents other than the statins. Exclusion: known comorbidities and use of metformin, digoxin, systemic corticosteroids, etc., within 4 weeks of screening	Primary: percent change in LDL-C from baseline to week 12. Secondary: percent change in apoB, non-HDL-C, total cholesterol, LDL particle number, HDL-C, HDL particle number, apolipoprotein A1, TG, VLDL particle number, high-intensity CRP from baseline to week 12	Bempedoic acid 120 mg or 180 mg added to stable statin therapy significantly reduced LDL-C compared to placebo
2	Ray et al. [14], 52 weeks, 2019, UK	CLEAR, harmony trial - randomized, double-blind, placebo-controlled, parallel-group, phase 3 trial	2230, test: 1488, placebo: 742	Bempedoic acid 180 mg P/O once daily or placebo for 12 weeks	Inclusion: hypercholesterolemic patients of both sexes aged 18-80 years, with ASCVD, and on maximally tolerated LMT, statins or others. Exclusion: concomitant use of simvastatin at average daily doses greater than 40 mg, concomitant use of PCSK9 inhibitors eGFR <30 ml/min/1.73 m2, and BMI ≥50 kg/m2	Primary: percent change from baseline to week 12 in LDL-C. Secondary: percent change in apoB, non-HDL-C, total cholesterol, LDL particle number, HDL-C, HDL particle number, apolipoprotein A1, TG, VLDL particle number, high-intensity CRP from baseline to week 12	Bempedoic acid added to maximally tolerated statin therapy did not lead to a higher incidence of overall adverse events than placebo and led to significantly lower HDL-C levels
3	Ballantyne et al. [15], 12 weeks, 2018 USA	Phase 3 multicenter, randomized, double-blind, placebo-controlled study	269, test: 181, placebo: 88	Bempedoic acid 180 mg P/O or placebo once daily 10 mg/day P/O for 12 weeks	Inclusion: history of statin intolerance, were on no more than low-dose statin therapy (which could also include no statin), and required additional LDL-C lowering. Exclusion: recent history of clinically significant CVD	Primary: percent change from baseline to week 12 in LDL-C. Secondary: percent changes from baseline to week 12 in non-HDL-C, total cholesterol, apoB, hsCRP, TG, and HDL-C	Bempedoic acid may provide an oral therapeutic option complementary to ezetimibe in statin-intolerant patients who require additional LDL-C lowering
4	Laufs et al. [16], 24 weeks, 2019, Germany	Phase 3, double-blind, placebo-controlled CLEAR, serenity study	345, test: 234, placebo: 111	Bempedoic acid 180 mg P/O or placebo once daily for 24 weeks	Inclusion: requiring statin therapy for primary or secondary prevention of CV events and history of requiring LMT based on local guidelines. Exclusion: recent MI/arrhythmia HbA1C >10%	Primary: percent change from baseline to week 12 in LDL-C. Secondary: percent changes from baseline to week 12 in non-HDL-C, total cholesterol, apoB, hsCRP, TG, and HDL-C	Bempedoic acid offers a safe and effective oral therapeutic option for lipid lowering in patients who cannot tolerate statins
5	Rosenson et al. [17], 12 weeks, 2019, USA	BANTING study, placebo-controlled, double-blind, randomized controlled study	424, test: 281, placebo: 143	Evolocumab 420 mg s.c. dosed on day 1, week 4, and week 8	Inclusion: ≥18 years of age with dyslipidemia and T2DM, HbA1c <10%, and taking a maximally tolerated dose of statin. Exclusion: recent MI	Primary: percent change from baseline to week 12 in LDL-C. Secondary: percent changes from baseline to week 12 in non-HDL-C, total cholesterol, apoB, hsCRP, TG, and HDL-C	Evolocumab significantly improved levels of other lipids and a reduction in LDL-C levels ≥50%
6	Lorenzatti et al. [18], 12 weeks, 2019, USA	BERSON, double-blind, 12-week, phase 3 study conducted in 10 countries	981, test: 657, placebo: 324	Evolocumab 140 mg subcutaneous Q2W, evolocumab 420 mg QM, placebo Q2W, or placebo QM for 12 weeks	Inclusion: ≥18 to ≤80 years of age with T2DM, HbA1c ≤10%, dyslipidemia, and on statin therapy. Exclusion: NYHA III or IV heart failure and MI	Primary: percent change from baseline to week 12 in LDL-C. Secondary: percent changes from baseline to week 12 in non-HDL-C, total cholesterol, apoB, hsCRP, TG, and HDL-C	In patients with T2DM and hyperlipidemia or mixed dyslipidemia on statin, evolocumab significantly reduced LDL-C and other atherogenic lipids, was well tolerated, and had no notable impact on glycaemic measures
7	Raal et al. [19], 12 weeks, 2012, USA	Reduction of LDL-C with PCSK9 inhibition in HeFH disorder (RUTHERFORD) study, phase 2, global, multicenter, double-blind, placebo-controlled trial	167, test: 111, placebo: 56	Evolocumab 350 mg and 420 mg subcutaneously every 4 weeks	Inclusion: men and women aged 18-75 years of age with HeFH despite at least 4 weeks of stable statin and other LLT before screening. Exclusion: homozygous FH elevated liver enzymes and heart failure	Primary: percent change from baseline to week 12 in LDL-C. Secondary: percent changes from baseline to week 12 in non-HDL-C, total cholesterol, apoB, hsCRP, TG, and HDL-C	Evolocumab administered every 4 weeks yielded rapid and substantial reductions in LDL-C in HeFH patients despite intensive statin use, with or without ezetimibe, with minimal adverse events and good tolerability
8	McKenney JM et al. [20], 12 weeks, 2012, USA	Double-blind, parallel-group, placebo-controlled randomized trial	183, test: 152, placebo: 31	Alirocumab (5 groups) 50 mg, 100 mg, 150 mg Q2W or 200 mg, 300 mg Q4W subcutaneously for 12 weeks	Inclusion: hypercholesterolemic patients of both sexes aged >18 years and on maximally tolerated statin dose. Exclusion: known hypersensitivity to monoclonal antibody and elevated liver enzymes and NYHA III or IV heart failure	Primary: percent change from baseline to week 12 in LDL-C. Secondary: percent changes from baseline to week 12 in non-HDL-C, total cholesterol, apoB, hsCRP, TG, and HDL-C	When added to atorvastatin, PCSK9 inhibition with alirocumab further reduces LDL-C by 40% to 72%. These additional reductions are both dose- and dosing-frequency-dependent
9	Kereiakes et al. [21], 52 weeks, 2015, USA	ODYSSEY COMBO I study, multicenter, phase 3, randomized (2:1 alirocumab vs. placebo), double-blind, 52-week trial	316, test: 209, placebo: 107	Alirocumab 75 mg every 2 weeks subcutaneously for 52 weeks	Inclusion: >18 years of age with coronary heart disease and receiving maximally tolerated stable daily dose of statins. Exclusion: known hypersensitivity to monoclonal antibody, elevated liver enzymes, and NYHA III or IV heart failure	Primary: percent change from baseline to week 24 in LDL-C. Secondary: percent changes from baseline to week 24 in non-HDL-C, total cholesterol, apoB, hsCRP, TG, and HDL-C	Alirocumab treatment achieved a significantly greater reduction in LDL-C versus placebo after 24 weeks with or without other LLT
10	Teramoto et al. [22], 52 weeks, 2016, Japan	ODYSSEY NIPPON, double-blind study, placebo-controlled randomized trial	163, test: 107, placebo: 56	Alirocumab (2 groups) 150 mg Q2W and Q4W subcutaneously for 52 weeks	Inclusion: >20 years of age with heFH and not achieved recommended LDL-C levels despite lipid-lowering therapies. Exclusion: known hypersensitivity to monoclonal antibody, elevated liver enzymes, and NYHA III or IV heart failure	Primary: percent change from baseline to week 12 in LDL-C. Secondary: percent changes from baseline to week 12 in non-HDL-C, total cholesterol, apoB, hsCRP, TG, and HDL-C	Hypercholesterolemic Japanese patients who tolerate only the lowest-strength dose statin or non-statin LLT can achieve robust LDL-C reduction with alirocumab 150 mg Q4W, in addition to their current LLT. Alirocumab 150 mg Q4W dosing was efficacious and generally well tolerated without new safety concerns
11	Ginsberg et al. [23], 78 weeks, 2016, USA	ODYSSEY HIGH FH study, multicenter, randomized, double-blind, placebo-controlled, phase 3 trial	107, test: 72, placebo: 36	Alirocumab 150 mg Q2W subcutaneously for 78 weeks	Inclusion: patients with heFH who are not adequately controlled with a maximally tolerated stable daily dose of statin for at least 4 weeks prior to the screening visit. Exclusion: not on a stable dose of LLT prior to the screening and currently taking a statin that is not simvastatin, atorvastatin, or rosuvastatin	Primary: percent change from baseline to week 24 in LDL-C. Secondary: percent changes from baseline to week 24 in non-HDL-C, total cholesterol, apoB, hsCRP, TG, and HDL-C	In patients with heFH and very high LDL-C baseline levels despite maximally tolerated statin ± other LLT, alirocumab 150 mg Q2W demonstrated significant reductions in LDL-C levels
12	Raal et al. [24], 52 weeks, 2020, South Africa	ORION 9 trial, phase 3, double-blind trial	481, test: 241, placebo: 240	Inclisiran sodium (at a dose of 300 mg, which corresponds to a dose of 284 mg of inclisiran free acid) or matching placebo, which were both administered as a 1.5-ml subcutaneous injection on days 1, 0, 270, and 450	Inclusion: subjects ≥18 years of age and history of HeFH. Exclusion: NYHA III or IV heart failure, active liver disease, and history of malignancy	Primary: percent change from baseline to day 510 in LDL-C. Secondary: percent changes from baseline to day 510 in non-HDL-C, total cholesterol, apoB, hsCRP, TG, and HDL-C	Inclisiran had significantly lower levels of LDL cholesterol than those who received placebo, with an infrequent dosing regimen and an acceptable safety profile
13	Ray et al. [25], 12 weeks, 2020, USA	ORION 10, randomized, double-blind, placebo-controlled, parallel-group, phase 3 trials	1561, test: 781, placebo: 780	Inclisiran (284 mg) or matching placebo, both administered as a 1.5-ml subcutaneous injection under blinded conditions on days 1, 90, 270, and 450	Inclusion: male or female subjects ≥18 years of age and history of ASCVD. Exclusion: NYHA III or IV heart failure, active liver disease, and treatment with PCSK9 inhibitors within 90 days of screening	Primary: percent change from baseline to day 510 in LDL-C. Secondary: percent changes from baseline to day 510 in non-HDL-C, total cholesterol, apoB, hsCRP, TG, and HDL-C	Among adults with HeFH, those who received inclisiran had significantly lower levels of LDL cholesterol than those who received placebo, with an infrequent dosing regimen and an acceptable safety profile
14	Ray et al. [25], 24 weeks, 2020, UK	ORION 11, randomized, double-blind, placebo-controlled, parallel-group, phase 3 trials	1617, test: 810, placebo: 807	Inclisiran (284 mg) or matching placebo, both administered as a 1.5-ml subcutaneous injection under blinded conditions on days 1, 90, 270, and 450	Inclusion: male or female subjects ≥18 years of age and history of ASCVD. Exclusion: NYHA III or IV heart failure, active liver disease, and treatment with PCSK9 inhibitors within 90 days of screening	Primary: percent change from baseline to day 510 in LDL-C. Secondary: percent changes from baseline to day 510 in non-HDL-C, total cholesterol, apoB, hsCRP, TG, and HDL-C	Among adults with HeFH, those who received inclisiran had significantly lower levels of LDL cholesterol than those who received placebo, with an infrequent dosing regimen and an acceptable safety profile

Percentage Reduction in LDL-C and Apolipoprotein-B

Table 2 shows the percentage change in LDL-C and apolipoprotein-B from baseline to 12 weeks for each drug included. Though each drug's administration duration varies, the cut-off assessment point of 12 weeks from baseline was chosen for this analysis for both LDL-C and apolipoprotein-B since it was common in all the included studies.

**Table 2:** Comparison of percentage reduction of LDL-C and apolipoprotein-B from baseline to week 12 and by the drugs included in the study Q2W: every 2 weeks, QM: every month, P/O: orally

Study number	Intervention drug details	Percentage reduction in LDL-C from baseline to 12 weeks	Percentage reduction in apolipoprotein-B from baseline to 12 weeks
1	Bempedoic acid 120 mg, 180 mg P/O, or placebo once daily in addition to ongoing statin therapy for 12 weeks [13]	Test group 1 bempedoic acid 120 mg: -17.3%. Test group 2 bempedoic acid 180 mg: -24.3%. Placebo group: -4.2%	Test group 1 bempedoic acid 120 mg: -15.0%. Test group 2 bempedoic acid 180 mg: -17.2%. Placebo group: -5.5%
2	Bempedoic acid 180 mg P/O once daily or placebo for 12 weeks [14]	Test group: -16.5%. Placebo group: -1.6%	Test group: -8.6%. Placebo group: -3.3%
3	Bempedoic acid 180 mg P/O or placebo once daily P/O for 12 weeks [15]	Test group: -28.5%. Placebo group: +5.0%	Test group: -19.3%. Placebo group: +4.7%
4	Bempedoic acid 180 mg P/O or placebo once daily for 24 weeks [16]	Test group: -23.6%. Placebo group: -1.3%	Test group: -15.5%. Placebo group: -0.2%
5	Evolocumab 420 mg s.c. dosed on day 1, week 4, and week 8 [17]	Test group: -54.3%. Placebo group: -1.1%	Test group: -40.3%. placebo group: +1.8%
6	Evolocumab 140 mg subcutaneous Q2W, evolocumab 420 mg QM, placebo Q2W, or placebo QM for 12 weeks [18]	Test group evolocumab 140 mg (Q2W): -64.7%. Placebo group 140 mg (Q2W): +7.1%. Test group evolocumab 420 mg (QM): -62.3%. Placebo group 140 mg (Q2W): +2.6%	Test group evolocumab 140 mg (Q2W): -53.9%. Placebo group 140 mg (Q2W): +3.2%. Test group evolocumab 420 mg (QM): -49.7%. Placebo group 140 mg (Q2W): -0.3%
7	Evolocumab 350 mg and 420 mg subcutaneously every 4 weeks [19]	Test group 1 evolocumab 350 mg (Q4W): -42.7%. Test group 2 evolocumab 420 mg (Q4W): -55.2%. Placebo group: +1.1%	Test group 1 evolocumab 350 mg (Q4W): -31.9%. Test group 2 evolocumab 420 mg (Q4W): -43.3%. Placebo group: +2.9%
8	Alirocumab (5 groups) 50 mg, 100 mg, 150 mg Q2W or 200 mg, 300 mg Q4W subcutaneously for 12 weeks [20]	Test group 1 alirocumab 50 mg: -39.6%. Test group 2 alirocumab 100 mg: -64.2%. Test group 3 alirocumab 150 mg: -72.4%. Test group 4 alirocumab 200 mg: -43.2%. Test group 5 alirocumab 300 mg: -47.7%. Placebo group: -5.1%	Test group 1 alirocumab 50 mg: -27.3%. Test group 2 alirocumab 100 mg: -48.1%. Test group 3 alirocumab 150 mg: -56.1%. Test group 4 alirocumab 200 mg: -28.7%. Test group 5 alirocumab 300 mg: -33.1%. Placebo group: +2.2%
9	Alirocumab 75 mg every 2 weeks subcutaneously for 52 weeks [21]	Test group: -46.3%. Placebo group: +1.1%	Test group: -34.7%. Placebo group: +3.5%
10	Alirocumab (2 groups) 150 mg Q2W and Q4W subcutaneously for 52 weeks [22]	Test group 1 alirocumab 150 mg (Q2W): -43.4%. Test group 2 alirocumab 150 mg (Q4W): -70.1%. Placebo group: -2.8%	Test group 1 alirocumab 150 mg (Q2W): -32.2%. Test group 2 alirocumab 150 mg (Q4W): -57.9%. Placebo group: -6.0%
11	Alirocumab 150 mg Q2W subcutaneously for 78 weeks [23]	Test group: -46.4%. Placebo group: -10.9%	Test group: -36.0%. Placebo group: -7.7%
12	Inclisiran sodium (at a dose of 300 mg or matching placebo, both administered as a 1.5-ml subcutaneous injection on days 1, 0, 270, and 450 [24]	Test group: -40.1%. Placebo group: +1.2%	Test group: -43.0%. Placebo group: +9.1%
13	Inclisiran (284 mg) or matching placebo, both administered as a 1.5-ml subcutaneous injection under blinded conditions on days 1, 90, 270, and 450 [25]	Test group: -51.3%. Placebo group: +1.0%	Test group: -44.8%. Placebo group: +1.7%
14	Inclisiran (284 mg) or matching placebo, both administered as a 1.5-ml subcutaneous injection under blinded conditions on days 1, 90, 270, and 450 [25]	Test group: -45.8%. Placebo group: +4.0%	Test group: -38.2%. Placebo group: +0.8%

Bempedoic Acid

A total of four studies involving bempedoic acid as a test drug with a total sample size of 2978 were included for this analysis. The percentage reduction in LDL-C and apolipoprotein-B among the four included studies ranges from 16.5-28.5% and 8.6-19.3% as compared to 1.3-4.2% and 0.2-5.5%, respectively. The maximum reduction in LDL-C (28.5%) and apolipoprotein-B (19.3%) was observed with bempedoic acid 180 mg per oral once daily for 12 weeks administered in statin-intolerant patients [15].

Evolocumab

Three studies with evolocumab with a total sample size of 1572 were included. The percentage reduction in LDL-C and apolipoprotein-B among the included studies ranged from 42.7-64.7% and 31.9-53.9%, respectively, in comparison with the placebo, where the parameters showed an increase in trend. The maximum reduction in LDL-C (64.7%) and apolipoprotein-B (53.9%) were noticed with evolocumab 140 mg administered subcutaneously every two weeks [18].

Alirocumab

Four studies with alirocumab with a sample size of 769 were included in this analysis. The percentage reduction in LDL-C and apolipoprotein-B among the included studies ranged from 39.6-72.4% and 27.3-57.9%, respectively, in comparison with placebo, 2.8-10.9% and 6.0-7.7%. The maximum reduction in LDL-C (72.4%) and apolipoprotein-B (56.1%) was noticed with alirocumab 150 mg subcutaneously administered every four weeks [20].

Inclisiran

ORION 9, ORION 10, and ORION 11 trials were included to represent inclisiran with a total sample size of 3659. The overall percentage reductions in LDL-C and apolipoprotein-B were in the range of 40.1-51.3% and 38.2-44.8%, respectively. The maximum reduction in LDL-C (51.3%) and apolipoprotein-B (44.8%) were noticed with the ORION 10 trial, where 284 mg inclisiran was given as a 1.5-ml subcutaneous injection on days 1, 90, 270, and 450 with a matching placebo [25].

Table 3 shows the total number of SAEs reported with the intervention drug in all the studies included. The significant SAEs were mentioned in the table, with none of them related to the drugs under study as reported in the articles. Hepatic carcinoma was more frequently reported to bempedoic acid and inclisiran, followed by stroke, and brain stem infarction was reported with evolocumab, alirocumab, and inclisiran. Serious cardiac adverse events like myocardial infarction and atrial fibrillation were reported with evolocumab and alirocumab. A rare SAE of leukocytoclastic vasculitis was reported with alirocumab. Though there were various SAEs reported with all the included drugs, they were not related to the intervention as per the causality assessment done by the investigators of the respective studies. Hence, the benefit-risk analysis should be considered while choosing an appropriate newer drug for treating hyperlipidemia.

**Table 3:** Compilation of SAEs reported to the drugs included in the study SAEs: serious adverse events

Study number	Intervention drug	Sample size (n)	No. of SAEs reported in the intervention group	Percentage of SAEs reported	Significant SAE reported
1	Bempedoic acid	134	1	0.7%	Ovarian adenoma [13]
2		2230	216	10%	Cardiac failure, hypertensive heart disease, myocardial infarction, lung cancer, ischemic cerebral infarction [14]
3		269	5	2%	Hepatic carcinoma [15]
4		345	14	4%	Chest pain, myocardial infarction, stroke [16]
5	Evolocumab	424	14	3%	Chronic obstructive pulmonary disease [17]
6		981	32	3%	Lacunar stroke, myocardial infarction [18]
7		167	2	1%	Atrial fibrillation [19]
8	Alirocumab	183	3	2%	Brain stem infarction [20]
9		316	26	8%	Leukocytoclastic vasculitis [21]
10		163	10	6%	Non-fatal myocardial infarction [22]
11		107	10	9%	Myocardial infarction [23]
12	Inclisiran	481	18	4%	Non-fatal stroke [24]
13		1561	175	11%	Hepatic carcinoma, pancreatic pseudocyst, ischemic stroke [25]
14		1617	181	11%	Hepatic carcinoma, non-fatal stroke, sepsis [25]

**Figure 2:**
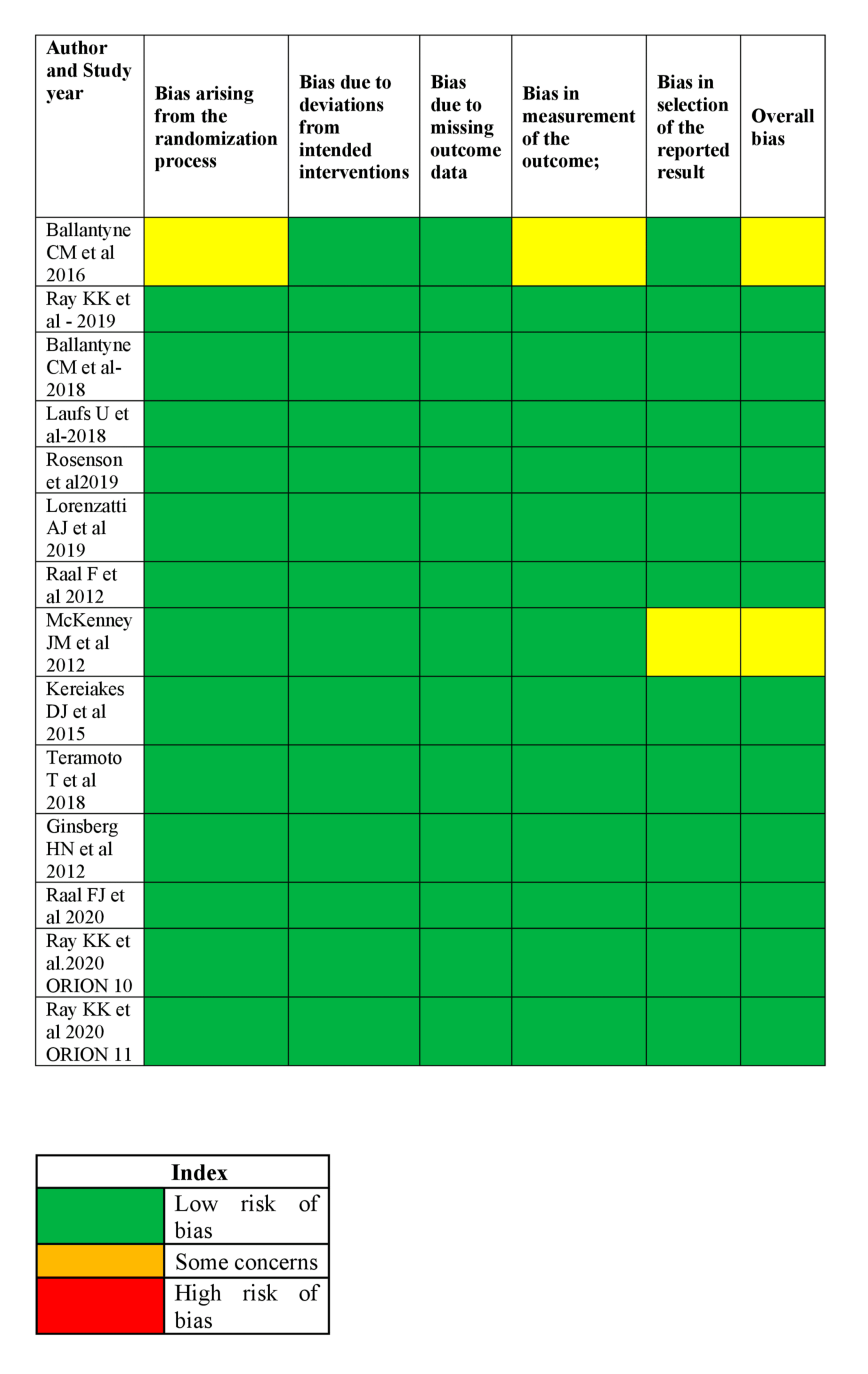
ROB of the included studies using Cochrane ROB assessment tool ROB: risk of bias

From this analysis, the maximum percentage reduction with LDL-C (72.4%) and apolipoprotein-B (57.9%) was observed with alirocumab. The maximum number of SAEs was reported with inclisiran (181, 11; 175, 11%), followed by bempedoic acid (216, 10%). Figure 2 shows the risk of bias assessment for the included studies. The majority of the studies had less risk of bias, as depicted in the image.

Discussion

Dyslipidemia poses a major risk for the development of ASCVDs. LDL-C and apolipoprotein-B are reliable predictors of cardiovascular risk. There are various drugs available to treat the condition, including statins and non-statin therapy. Some of the newly approved drugs are bempedoic acid, inclisiran, and PCSK9 inhibitors. This review analyzed the LDL-C- and apolipoprotein-B-lowering capacity of bempedoic acid, inclisiran, evolocumab, and alirocumab. Table 4 illustrates the detailed pharmacokinetics and pharmacodynamic data for the abovementioned drugs. 

**Table 4:** Pharmacokinetics and pharmacodynamic parameters of the drugs included in the study US FDA: United States Food and Drug Administration, ACL: adenosine triphosphate-citrate lyase, LDL-C: low-density lipoprotein cholesterol, HeFH: heterozygous familial hypercholesterolemia, ESRD: end-stage renal disease, PCSK9: proprotein convertase subtilisin kexin type 9, LDL: low-density lipoprotein, HoFH: homozygous familial hypercholesterolemia, siRNA: small interfering RNA, NIL: none, Cmax: maximum concentration, AUC: area under the curve [26]

S. no	Properties	Bempedoic acid	Evolocumab	Alirocumab	Inclisiran
1.	Group	Hypolipidemic drug	Hypolipidemic drug	Hypolipidemic drug	Hypolipidemic drug
2.	Category of drug	Alpha, omega-dicarboxylic acid	Monoclonal antibody	Monoclonal antibody	siRNA
3.	Manufacturer name	Esperion Therapeutics, Inc.	Amgen	Regeneron and Sanofi	Alnylam pharmaceuticals
4.	Year of US FDA approval	2020	2015	2015	2021
5.	Mechanism of action	ACL inhibitor that lowers LDL-C by inhibition of cholesterol synthesis in the liver	PCSK9 inhibitor antibody. Preventing PCSK9-mediated LDL receptor degradation	PCSK9 inhibitor antibody. Preventing PCSK9-mediated LDL receptor degradation	Double-stranded siRNA, directs catalytic breakdown of mRNA for PCSK9, increases LDL-C receptor recycling and expression on the hepatocyte cell surface, which increases LDL-C uptake and lowers LDL-C levels in the circulation
6.	Indication as per US FDA label	To reduce LDL-C in adults with primary hyperlipidemia, HeFH, combination with other LDL-C-lowering therapies or alone. To reduce the risk of myocardial infarction and coronary revascularization in adults who are unable to take recommended statin therapy	To reduce LDL-C in adults with primary hyperlipidemia, HeFH, combination with other LDL-C-lowering therapies or alone. To reduce the risk of myocardial infarction and coronary revascularization in adults who are unable to take recommended statin therapy. Adjunct to diet and other LDL-lowering therapies with HoFH	To reduce LDL-C in adults with primary hyperlipidemia, HeFH, combination with other LDL-C-lowering therapies or alone. To reduce the risk of myocardial infarction and coronary revascularization in adults who are unable to take recommended statin therapy	To reduce LDL-C in adults with primary hyperlipidemia, HeFH, combination with other LDL-C-lowering therapies or alone. To reduce the risk of myocardial infarction and coronary revascularization in adults who are unable to take recommended statin therapy
7.	Route of administration	Per oral (tablets)	Subcutaneously	Subcutaneously	Subcutaneously
8.	Dosage and frequency of administration	180 mg orally once daily	140 mg every 2 weeks or 420 mg once monthly	75 mg once every 2 weeks administered subcutaneously (or) 300 mg once every 4 weeks (monthly)	284 mg administered as a single subcutaneous injection initially, again at 3 months, and then every 6 months in combination with maximally tolerated statin therapy
9.	Dosage forms and strengths	Tablets: 180 mg	Injection: 140 mg/mL solution in a single-use prefilled syringe. Injection: 140 mg/mL solution in a single-use prefilled autoinjector. Injection: 420 mg/3.5 mL solution in a single-use system (on-body infuser with prefilled cartridge)	Injection: 75 mg/mL or 150 mg/mL solution in a single-dose pre-filled pen. Injection: 75 mg/mL or 150 mg/mL solution in a single-dose pre-filled syringe	Injection: 284 mg/1.5 mL (189 mg/mL) in a single-dose prefilled syringe
10.	Half-life volume of distribution plasma protein binding	21 + 11 hours, 18 L, 99.3%	11 to 17 days 3.3 L	17 to 20 days 0.04 to 0.05 L	9 hours, 500 L, 87%
11.	Metabolism	Through metabolism of the acyl glucuronide	Two elimination phases: at low concentrations - saturable binding to target PCSK9. At higher concentrations - non-saturable proteolytic pathway	Degrade to small peptides and individual amino acids	By nucleases to shorter nucleotides of varying length
12.	Route of elimination	Kidney	Two elimination phases: at low concentrations - saturable binding to target PCSK9. At higher concentrations - non-saturable proteolytic pathway	Two elimination phases: at low concentrations - saturable binding to target PCSK9. At higher concentrations -non-saturable proteolytic pathway	Kidney
13.	Adverse drug reactions	Upper respiratory tract infection, muscle spasms, hyperuricemia, back pain, abdominal pain or discomfort, bronchitis, pain in extremity, anemia, and elevated liver enzymes	Nasopharyngitis, upper respiratory tract infection, influenza, back pain, and injection site reactions	Nasopharyngitis, injection site reactions, and influenza	Injection site reaction, arthralgia, urinary tract infection, diarrhea, bronchitis, pain in extremities, and dyspnea
14.	Contraindications	History of a serious hypersensitivity reaction to bempedoic acid	History of a serious hypersensitivity reaction to evolocumab	History of a serious hypersensitivity reaction to alirocumab	NIL
15.	Drug interactions	Avoid concomitant use with simvastatin and pravastatin	NIL	NIL	NIL
16.	Indication of renal failure	No dosage adjustment is necessary in patients with mild or moderate renal impairment. Not studied in ESRD patients	No dose adjustment is needed	No dose adjustment is needed	Increase in inclisiran Cmax and AUC in mild, moderate, or severe renal impairment
17.	Indication of liver failure	No dose adjustment is necessary for mild or moderate hepatic impairment (Child-Pugh A or B). Not studied in severe hepatic impairment	No dose adjustment is necessary for mild or moderate hepatic impairment. Not studied in severe hepatic impairment	No dose adjustment is necessary for mild or moderate hepatic impairment. Not studied in severe hepatic impairment	In moderate hepatic impairment, baseline PCSK9 levels were lower and reductions in LDL-C were less than those observed in patients with normal hepatic function. Not studied in patients with severe hepatic impairment
18.	Status in pregnancy	May cause fetal harm when administered to pregnant patients based on the mechanism of action. Discontinue bempedoic acid when pregnancy is recognized unless the benefits outweigh the risks	Effect of pregnancy on evolocumab pharmacokinetics - not studied	No available data on use in pregnant women	May cause fetal harm when administered to pregnant patients based on the mechanism of action. To be discontinued when pregnancy is recognized
19.	Status in lactation	Breastfeeding is not recommended with bempedoic acid	No information regarding the presence of evolocumab in human milk	No information regarding the presence of alirocumab in human milk	No information regarding the presence of alirocumab in human mil

From our analysis of four studies with bempedoic acid, the maximum LDL-C and apolipoprotein-B reductions noticed with bempedoic acid were 28.5% and 19.3%, respectively. In a study conducted by Goldberg et al. [27], with a sample size of 779, where bempedoic acid was added to maximally tolerated statin therapy, the percentage reduction of LDL-C from baseline to 12 weeks was 15.4%, which was compared to the studies we had included in this analysis. A study was conducted by Laufs et al. [28] to assess the efficacy and safety of bempedoic acid in patients not receiving statins. The percentage reduction in LDL-C from baseline to 12 weeks was 26.5% [28]. This study evokes a thought on assessing the effectiveness of bempedoic acid monotherapy in dyslipidemic patients. This approach can be manifested with the help of conducting various trials on bempedoic acid as monotherapy.

Out of three studies with evolocumab, the maximum percentage reduction of LDL-C and apolipoprotein-B from baseline to 12 weeks observed were 64.7% and 53.9%, respectively. This was observed in a study conducted by Lorenzatti et al. [18] with evolocumab 140 mg given every two weeks subcutaneously, where they included patients with dyslipidemia and type 2 diabetes mellitus and statin therapy. The LDL-C reduction was almost similar to a study conducted by Sabatine et al. [29], where the percentage reduction of LDL-C was 61%, and 4465 patients were randomized to either evolocumab 140 mg every two weeks or 420 mg monthly plus standard therapy or standard therapy alone. In a study by Hao et al. [30] investigating the application of evolocumab with standard therapy versus standard therapy alone in patients with high-risk acute coronary syndrome, LDL-C was reduced by 83.88% and apolipoprotein-B by 63.89% in the evolocumab with standard therapy group, which was higher compared to the reductions observed in the studies included in this analysis. This reflects the emerging role of evolocumab in dyslipidemia with potential to control lipid profile as well as cardiovascular risk.

The greater reduction of LDL-C and apolipoprotein-B with alirocumab was 72.4% and 57.9%, respectively. It was observed in a study by McKenney et al. [20], which was a double-blind, parallel-group, placebo-controlled randomized trial with a sample size of 183. The study included dyslipidemic patients who were on atorvastatin 10 mg/20 mg/40 mg daily. In a study by Cannon et al. [31] to assess the efficacy and safety of alirocumab vs. placebo in patients (n=720) with a high risk of CVD, the percentage reductions in LDL-C and apolipoprotein-B were 51.2% and 38.7% from baseline to 12 weeks, which was less compared to our included studies, though the sample size was larger. A study assessed the long-term safety and efficacy of alirocumab in patients with HeFH (n=985), the percentage reduction in LDL-C at week 8 and week 96 was 44.2% and 47.9%, respectively [32]. These results were quite contradictory to the studies included in our analysis, where the percentage reduction in LDL-C was in a lower range event at 96 weeks.

Inclisiran reduced LDL-C and apolipoprotein-B to a maximum of 51.3% and 44.8%, respectively. This was observed in a study done by Ray et al. [24] with a sample size of 1561 patients with ASCVD and uncontrolled dyslipidemia despite statin therapy, where inclisiran 284 mg or matching placebo was administered as a 1.5 ml subcutaneous injection on days 1, 90, 270, and 450. In a study [33], inclisiran was found to decrease the LDL-C from baseline to 12 weeks by 38% and 45% in patients with added statins. These results were almost in concordance with our analyzed studies. In another study by Luo et al. [34] in Chinese patients, inclisiran 1000 mg or 300 mg single dose subcutaneous injection reduced the LDL-C from baseline to 12 weeks by 49.6% and 58.3%, respectively. Though the half-life of inclisiran is short, a single dose injection could effectively reduce the LDL-C. The LDL-C lowering effects in the Chinese population were similar to the other global clinical trials, as observed in our analysis, where the range of LDL-C reduction was 40.1-51.3%, Thus, inclisiran is emerging as a prospective lipid-lowering agent in all the ethnic populations. Thus, the maximum percentage reduction with LDL-C (72.4%) and apolipoprotein-B (57.9%) was observed with alirocumab. The potential reason could be due to the longer duration of action of alirocumab (17 to 20 days).

The SAEs reported were greater with bempedoic acid 216 (10%) such as myocardial infarction, hepatic carcinoma, ovarian adenoma, etc. This could be due to a larger sample size (n=2230) or due to the inclusion of patients with ASCVD [14]. Also, bempedoic acid is given as an oral preparation, increasing the chances of adverse effects. Bempedoic acid is a prodrug that requires activation by acyl-CoA-synthetase-1. There is a reduced maximum concentration and area under the curve of bempedoic acid in patients with mild to moderate hepatic impairment. However, this variation is not clinically significant and does not result in reduced efficacy [27]. Followed by bempedoic acid, inclisiran had the maximum SAEs [25] of 181 (11%) and 175 (11%) such as non-fatal stroke and hepatic carcinoma. The possible reasons could be due to a large sample size of 1617 and 1561, including patients with a history of ASCVD since the reported SAE was myocardial infarction. To avoid such SAEs, more preliminary trials could be conducted to assess the change in LDL-C in patients without other comorbid conditions, especially CVD. This will be helpful to correlate whether the LDL-C lowering effect has an effective impact on long-term cardiovascular risk prevention employing prospective cohort studies.

In all the included bempedoic acid studies in this analysis, the muscle-related adverse effects are less compared to the control group. Bempedoic acid inhibits the ATP citrate lyase, increases the uptake of LDL particles by the liver, and reduces the LDL-C in the blood. It is a prodrug and requires activation by acyl-CoA-synthetase-1, which is present only in the liver. Hence, the muscle-related adverse effects such as myopathy and rhabdomyolysis are less with bempedoic acid compared to statins, although both drugs act through the same pathway [35]. Thus, bempedoic acid might prove to be a beneficial alternative in statin-intolerant patients.

Considering most of the patients with hypercholesterolemia are in the older age group with multiple comorbidities, including renal dysfunction, PCSK9 inhibitors like evolocumab and alirocumab would be helpful in such cases, as monoclonal antibodies are relatively safe in renal dysfunction. This could be an advantage of PCSK9 inhibitors in dyslipidemic patients with chronic kidney disease [36].

In comparison with PCSK9 inhibitors, which are also administered as subcutaneous injections, inclisiran provides more sustained PCSK9 suppression by silencing the mRNA and also increases LDL-C uptake by enhancing the LDL-C receptor recycling in the liver, which produces sustained lower blood LDL-C levels. It is also a longer-acting agent with less frequent dosing, i.e., the initial dose followed by every three monthly and every six monthly injections. This could be convenient for patients who are not able to take oral medications daily [37]. Among the included drugs in this analysis, LDL-C and apolipoprotein-B reduction were comparatively lower with bempedoic acid (LDL-C: 16.5-28.5%; apolipoprotein-B: 8.6-19.3%) than with evolocumab, alirocumab, and inclisiran.

Given that bempedoic acid is the only oral drug among the other four included drugs, there is a possibility of increased patient compliance in the long run with this drug as it is convenient for administration. Nevertheless, the other three drugs, including PCSK9 inhibitors, though administered subcutaneously, will be advantageous because of their less frequent administration. Thus, the choice of drug therapy has to be individualized according to a particular patient’s condition and preference. To assess the compliance of these drugs in patients with hypercholesterolemia, the scientific platform anticipates more studies in the near future.

## Conclusions

Dyslipidemia is a primary risk factor for CVDs and can significantly worsen the prognosis if left untreated. Among lipid parameters, LDL-C and apolipoprotein-B are crucial predictors of cardiovascular risk. This systematic review evaluates the efficacy of recently approved drugs for dyslipidemia, including bempedoic acid, evolocumab, alirocumab, and inclisiran, in reducing LDL-C and apolipoprotein-B levels. The maximum percentage reduction of LDL-C and apolipoprotein-B was observed with alirocumab. However, this cannot be taken as a concluding remark as many other factors, such as inclusion criteria, comorbid conditions, and baseline lipid profile parameters, vary from one study to another. Hence, informed clinical decision-making with individual patient profiles is necessary to decide on better therapy among the available newer drugs.

## Appendices

**Table 5:** Search strategies utilized in this review

S. No.	Drug	Search terms utilized
1.	Bempedoic acid	((bempedoic acid [MeSH Terms]) AND (dyslipidemia [MeSH Terms])) OR (hyperlipidemia [MeSH Terms])
2.	Evolocumab	((Evolocumab[Mesh]) AND (Dyslipidemias[Mesh] OR Hyperlipidemias[Mesh] OR Hypercholesterolemia[Mesh] OR Lipid Metabolism Disorders[Mesh]))
3.	Alirocumab	((Alirocumab [Mesh]) AND (Dyslipidemias [Mesh] OR Hyperlipidemias [Mesh] OR Hypercholesterolemia [Mesh] OR Lipid Metabolism Disorders [Mesh]))
4.	Inclisiran	((Inclisiran[Mesh]) AND (Dyslipidemias[Mesh] OR Hyperlipidemias[Mesh] OR Hypercholesterolemia[Mesh] OR Lipid Metabolism Disorders[Mesh]))
